# Photopharmacology of Ion Channels through the Light of the Computational Microscope

**DOI:** 10.3390/ijms222112072

**Published:** 2021-11-08

**Authors:** Alba Nin-Hill, Nicolas Pierre Friedrich Mueller, Carla Molteni, Carme Rovira, Mercedes Alfonso-Prieto

**Affiliations:** 1 Departament de Química Inorgànica i Orgànica (Secció de Química Orgànica) and Institut de Química Teòrica i Computacional (IQTCUB), Universitat de Barcelona, 08028 Barcelona, Spain; albanin@ub.edu (A.N.-H.); c.rovira@ub.edu (C.R.); 2 Institute for Advanced Simulations IAS-5 and Institute of Neuroscience and Medicine INM-9, Computational Biomedicine, Forschungszentrum Jülich, 52425 Jülich, Germany; nic.mueller@fz-juelich.de; 3 Faculty of Mathematics and Natural Sciences, Heinrich-Heine-University Düsseldorf, Universitätsstr. 1, 40225 Düsseldorf, Germany; 4 Physics Department, King’s College London, London WC2R 2LS, UK; carla.molteni@kcl.ac.uk; 5 Institució Catalana de Recerca i Estudis Avançats (ICREA), 08020 Barcelona, Spain; 6 Cécile and Oskar Vogt Institute for Brain Research, University Hospital Düsseldorf, Medical Faculty, Heinrich Heine University Düsseldorf, 40225 Düsseldorf, Germany

**Keywords:** photopharmacology, photoswitchable ligands, ion channels, voltage-gated ion channels, ligand-gated ion channels, homology modeling, molecular docking, molecular dynamics, enhanced sampling

## Abstract

The optical control and investigation of neuronal activity can be achieved and carried out with photoswitchable ligands. Such compounds are designed in a modular fashion, combining a known ligand of the target protein and a photochromic group, as well as an additional electrophilic group for tethered ligands. Such a design strategy can be optimized by including structural data. In addition to experimental structures, computational methods (such as homology modeling, molecular docking, molecular dynamics and enhanced sampling techniques) can provide structural insights to guide photoswitch design and to understand the observed light-regulated effects. This review discusses the application of such structure-based computational methods to photoswitchable ligands targeting voltage- and ligand-gated ion channels. Structural mapping may help identify residues near the ligand binding pocket amenable for mutagenesis and covalent attachment. Modeling of the target protein in a complex with the photoswitchable ligand can shed light on the different activities of the two photoswitch isomers and the effect of site-directed mutations on photoswitch binding, as well as ion channel subtype selectivity. The examples presented here show how the integration of computational modeling with experimental data can greatly facilitate photoswitchable ligand design and optimization. Recent advances in structural biology, both experimental and computational, are expected to further strengthen this rational photopharmacology approach.

## 1. Introduction

**Figure 1:**
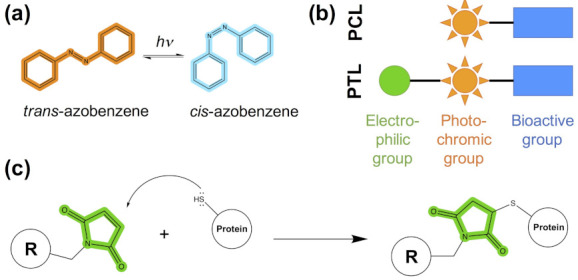
Chemical toolbox for the design of photoswitchable ligands. (**a**) Chemical structure of azobenzene, the most commonly used photochromic group, showing its *trans-cis* photoisomerization. (**b**) Modular design of photoswitchable ligands, either soluble photochromic ligands (PCLs) or photochromic tethered ligands (PTLs). (**c**) Covalent bond formation between the typical tethering protein residue, cysteine (shown here by its sidechain thiol group) and a common electrophile group included in PTLs, maleimide (colored in green); the rest of the PTLs are represented as a substituent R.

Photopharmacology (also known as optopharmacology) is a discipline that aims at regulating the activities of biological systems with light. Light-controlled modulation can be accomplished with photoswitchable compounds [1,2,3,4]. Such molecules contain a bioactive ligand coupled to a photochromic group that, upon irradiation, causes bond isomerization or formation. For instance, the most commonly used photochromic group, azobenzene [5], isomerizes between *trans* and *cis* configurations (Figure 1a). This results in changes in both length and dipole moment that can affect the shape and chemical complementarity of the photoswitchable ligand with the protein binding pocket. When the two forms of the photoswitchable ligand have different binding preferences and/or differentially regulate protein function, optical control is achieved. Thereby, irradiation with light of the appropriate wavelength can turn protein activity on or off with high temporal and spatial resolutions.

Photoswitchable ligands have been widely applied to the field of ion channels, because the picosecond timescale of the photochromic group transition upon irradiation is faster than the timescale of ion flow across the neuronal membrane. Among other applications, photopharmacology has been used to study ion channel properties and kinetics, the regulation of neuronal circuits and the control of animal responses, such as heartbeat, pain, vision and behavior [4]. Two main types of photoswitches have been used: freely diffusible photochromic ligands (PCLs) and photoswitchable tethered ligands (PTLs). Their design is modular (Figure 1b), containing a ligand known to regulate protein function (bioactive group) connected to a photochromic group (e.g., azobenzene). In the case of PTLs, they additionally contain an electrophilic group that binds covalently to an amino acid with nucleophilic properties near the binding site (typically cysteine; see Figure 1c). Although this nucleophilic residue can be naturally present in the target protein, in most cases the reactive cysteine is introduced by site-directed mutagenesis (i.e., optochemical genetics [6]). In addition to PCLs and PTLs, photopharmacological applications to ion channels can also employ photocaged ligands, which contain a protecting group (i.e., the cage) that is cleaved upon light irradiation, resulting in a rapid release of the bioactive molecule (e.g., the neurotransmitter). However, their design has been extensively reviewed in references [4,7,8,9,10] and thus will not be considered here.

**Figure 2:**
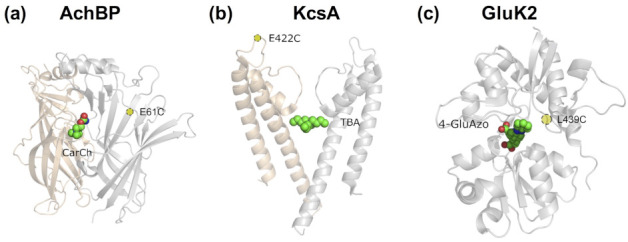
Crystallographic structures used for rational structure-based design of photoswitchable ligands (**a**,**b**) or its validation (**c**). (**a**) Structure of the acetylcholine binding protein (AchBP) bound to carbamylcholine (CarCh), PDB code 1UV6 [15]. (**b**) Structure of the KcsA potassium channel with the pore blocker tetrabutylammonium (TBA) bound in the intracellular site below the selectivity filter, PDB code 2BOB [18]. (**c**) Structure of the ligand binding domain (LBD) of the kainate receptor GluK2 in complex with 4-glutamyl-azobenzene (4-GluAzo), PDB code 4H8I [19]. For the sake of clarity, only one or two subunits of the oligomeric proteins are shown in cartoon representation and colored either in gray or apricot. Ligands are displayed in space-filling representation, with C, O and N atoms colored in green, red and blue, respectively. The position of the cysteine residue serving as covalent attachment point for the PTL is highlighted with a yellow circle.

The first ion channel to be photomodulated was the nicotinic acetylcholine receptor (nAchR). Both PCLs and PTLs were developed [11,12], consisting of a known nAchR ligand linked to a photoswitchable azobenzene group and, for PTLs, also coupled to a benzylic bromide (i.e., the electrophile for Cys tethering). In the *trans* form, the photoswitchable ligand was able to modify the receptor activity, whereas isomerization to *cis* upon UV light irradiation turned off the modulatory effect of the photoswitch. Although at the time molecular biology techniques were still in their infancy, two fortunate coincidences contributed to this first success story. On the one hand, nAChR is highly expressed in the electroplaques of electric eels. On the other, even without sequence knowledge, it was known that treatment with a disulfide reducing agent generated a free cysteine residue that allowed the covalent conjugation of tethered ligands [13]. Despite this remarkable achievement, it was still unexplained why some of the PTLs, designed based on nAChR agonists, acted instead as light-modulated antagonists [11], i.e., why the addition of the azobenzene group was changing the ligand pharmacological properties. More than thirty years later, experimental determination of the first crystal structures of the snail acetylcholine binding protein (AchBP, the soluble counterpart of the ligand binding extracellular domain of nAChR) gave a hint of the molecular basis of this change in activity (Figure 2a). The degree of closure of the so-called loop C over the binding site is correlated with the agonist (closed loop) or antagonist (open loop) activity of the cholinergic ligands [14,15,16,17]. This demonstrates that, although a photoswitchable ligand design can be successfully achieved with only ligand structure–activity relationship data, structural knowledge of the target receptor or ion channel can greatly facilitate such a task [2].

The next generation of photoswitchable ligands was developed in the early 2000s thanks to the resolution of the crystal structures of a voltage-gated potassium channel and an ionotropic glutamate receptor. Based on the KcsA crystal structure solved in the presence of tetraethylammonium (TEA)-like pore blockers (Figure 2b) [18,20], light-control blockage of the homologous Shaker potassium channel [21,22] was achieved. A Cys mutation was introduced near the extracellular TEA binding site (E422C mutant) and a PTL was synthetized consisting of a Cys-reactive maleimide group, an azobenzene photoswitch and quaternary ammonium (MAQ). *Trans*-MAQ extends from the tethering site to reach the ammonium binding site in the pore, whereas *cis*-MAQ is too short to do so. Similarly, the X-ray structure of the soluble ligand binding domain (LBD) of the kainate receptor [23] was used to design photoswitchable ligands targeting this receptor. Namely, a PCL (consisting of the glutamate agonist and an azobenzene photoswitch, 4-GluAzo) [24] and a PTL (composed of a Cys-reactive maleimide, an azobenzene photoswitch and the glutamate ligand, MAG) [25] were developed. The latter covalently attaches to a light-modulated glutamate receptor (LiGluR) containing an L439C mutation. Moreover, the structure-based PCL design was later validated by solving the crystal structure of 4-GluAzo bound to the GluK2 LBD [19] (Figure 2c). The clamshell-like structure of the LBD showed a closed state, similar to other agonist-bound LBD structures and in contrast with the open state observed in the antagonist-bound structures [23]. Therefore, the design of photoswitchable ligands with the desired activity requires not only information about the protein structure, but also about the dynamical rearrangements occurring after ligand binding [2].

Unfortunately, experimental structures for the (voltage-gated and ligand-gated) ion channels involved in neurotransmission are still scarce, despite recent advances in structural biology tools, in particular cryo-electron microscopy (cryo-EM). To fill this gap, computational modeling can be used to generate structural models for these channels. Moreover, computer-aided drug design techniques (either ligand- or structure-based) can be applied to the design of photoswitchable ligands. Indeed, a recent computational study showed that a large number of bioactive molecules can be susceptible of azologization (i.e., fragment replacement by an isosteric azobenzene group to make the drug photoswitchable) [26]. In other words, a computational microscope, a term coined by the late Klaus Schulten to describe the use of modeling and simulations to study protein function and dynamics [27], can also shed light on the photopharmacology field.

For a systematic review of all the PCLs and PTLs available to date, we refer the reader to several excellent published reviews on photopharmacology or optochemical genetics [1,2,3,4,6,10,28,29,30,31,32,33,34]. Here we focus on photopharmacological applications targeting ion channels in which structure-based computational methods were applied, in combination with experimental approaches. We start with a short theoretical description of the computational methods used for photopharmacology so far. Then, we present some of the applications published in the literature (Tables S1–S4), where computational methods have been used to rationally design and optimize photoswitchable ligands, explain their observed effect on ion channel activity and/or identify possible tethering positions for Cys mutation. We have classified such studies depending on whether the photoswitchable ligand targets voltage- or ligand-gated ion channels (VGICs and LGICs, respectively). The chemical structures of all the PCLs and PTLs discussed in the text are shown in Figures S1–S4. To the best of our knowledge, most ion channel photopharmacology studies integrating computational methods have been carried out on azobenzene-based photoswitchable ligands.

## 2. Computational Modeling

**Figure 3:**
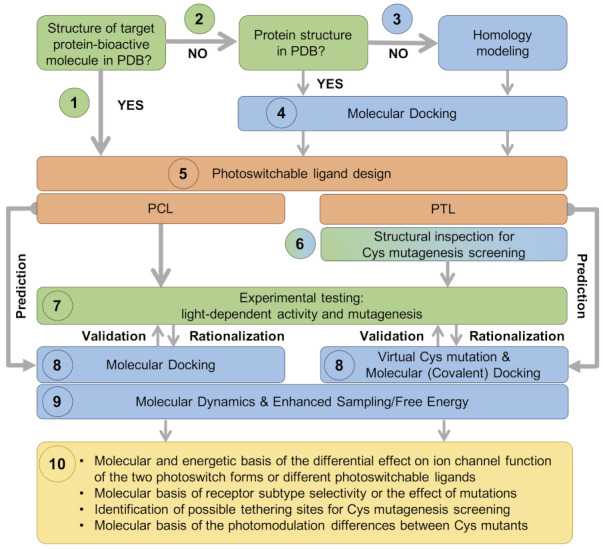
Proposed workflow for rational, structure-based design of photoswitchable ligands. The design step is indicated in orange, the experimental steps in green and the computational steps in blue. Ligand-based data (such as structure–activity relationship data or information about tethered, non-photoswitchable ligands), though not shown here, can also be integrated in the design step. Some of the possible information outcomes of this integrative computational-experimental workflow are listed in the yellow box.

Ion channels are oligomeric proteins, in which several subunits assemble to form the functional channel (see Section 3 and Section 4 below). The large number of VGIC families, as well as receptor (sub)types for LGICs, gives rise to a large number of possible combinations. Moreover, each of these ion channels can adopt different functional states (open, closed, desensitized or inactivated) and their activities can be regulated by a myriad of ligands (agonists and antagonists, as well as pore blockers and allosteric modulators). Unlike the examples mentioned in the Introduction [21,22,25], an experimental structure of the ion channel of interest (in the relevant functional state and in complex with the ligand used as a bioactive group of the PCL or PTL) may not be available. This structural gap can be filled by structure-based computational approaches, such as those included in Figure 3. In the following, we mention some basic ideas underlying these methodologies; a full description of these computational methods is beyond the scope of this review, and thus we refer the reader to the excellent advanced reviews cited below.

Homology modeling generates structural models of the target protein based on its sequence and the experimental structure of a homologous protein (the so-called template). The quality of the homology model depends on the sequence identity between the target and template proteins, with 35% sequence identity being considered the minimum threshold for homologous membrane proteins to have similar 3D structures [35,36]. Moreover, structural rearrangements occur upon ligand binding and opening/closing of the ion channel pore; thus, it is recommended to choose a template structure not only with the highest sequence identity, but also captured in the appropriate functional state.

Molecular docking aims at predicting protein–ligand interactions (in the case at hand, between the ion channel of interest and the photoswitchable ligand) [37]. Docking can be performed using either an experimental structure or a homology model of the ion channel of interest. If information about the putative ligand binding site is already available, it can be incorporated into the computation protocol to guide the docking (i.e., information-driven docking). This includes the structural information of the bioactive part of the photoswitch bound to the ion channel, as well as mutagenesis data, indicating which residues are likely to be interacting with the bioactive molecule and/or the photoswitch. Otherwise, a blind docking approach, in which all possible binding pockets on the protein surface are explored, can be the method of choice. In most docking protocols, the ligand is considered flexible, whereas the receptor structure can be treated as rigid or flexible. In the latter case, only amino acids surrounding the binding site are usually allowed to move in order to model the induced fit effects. In the case of PTLs, the covalent bond between the tether and the reactive Cys can be modeled by using either a positional constraint (limiting the movement of the electrophile group within a certain sphere from the reactive Cys) or a distance restraint between the two groups. Interestingly, molecular docking (and in general computational modeling) allows one not only to model the photoswitch isomer that preferentially binds to the target protein, but also the other isomer, providing molecular insights into the light-modulated changes in ion channel activity. Moreover, virtual mutations can be introduced in the target protein structure to model the changes in photoswitch binding upon site-directed mutagenesis or when using different ion channel subtypes.

Homology modeling and molecular docking can provide static structures of the target protein in complex with the photoswitch. Additionally, Monte Carlo (MC) or molecular dynamics (MD) simulations can be performed. Therewith, the target protein–photoswitchable ligand complex is embedded in a lipid bilayer mimicking the physiological membrane environment and, upon equilibration/minimization, several configurations of the system are sampled, thus providing a dynamical picture. MD models the physical movements of the system as a function of time by solving Newton’s equations of motion, whereas MC generates an ensemble of configurations according to the corresponding Boltzmann distribution. Such simulations allow one to further characterize the conformational rearrangements occurring upon ligand binding and their connection with ion conduction [38,39,40,41,42,43,44]. In combination with enhanced sampling methods and/or free energy calculations, MD can also provide an estimate of the ligand affinity (e.g., binding energy differences between the two forms of the photoswitch or between two photoswitchable ligands), as well as molecular insights into the energetic determinants of binding (e.g., to identify the most suitable position for introducing a reactive Cys for PTL covalent attachment).

Nonetheless, such computational methodologies also have limitations. The quality of the homology models may not be sufficient for an accurate prediction of PCL or PTL binding, especially if the sequence identity of the target protein with the template is low (i.e., below 35%) and/or the structural changes occurring upon ligand binding are not similar to those captured in the available experimental structures. Whereas molecular docking can only model small rearrangements in the protein side chains to accommodate the ligand, molecular dynamics combined with enhanced sampling techniques can be attempted to simulate further protein conformational rearrangements. However, it is still challenging to simulate large structural changes in proteins due to the long time scales of these processes and the limited quality of the (protein and ligand) force fields. Therefore, a close interplay of these in silico studies with in vitro and in vivo assays is key for successful photoswitchable ligand design. On the one hand, the experimental data are used to guide the calculations and to validate the computational models. On the other, the computational data provide a molecular explanation of the photoswitch-mediated modulation and help design modifications of the photoswitchable ligand and/or mutations of the ion channel (including Cys mutants for tethering) that can be tested experimentally. In other words, structure-based computational methods provide insights complementary to experiments that link the molecular details of the photoswitchable ligands to the experimentally measured macroscopic effects.

Figure 3 shows a possible workflow for the rational, structure-based designing of photoswitchable ligands, based on the following steps:

(1) In the most straightforward case, a search in the Protein Data Bank (PDB) yields an experimental structure of the target protein in complex with the bioactive molecule to be used as a basic module of the photoswitchable ligand. A structure of the target protein bound to a similar molecule (in terms of chemical structure and activity) or a structure of a homologous protein–ligand complex can also be used as a surrogate, as demonstrated by the examples mentioned in the Introduction.
(2) In the absence of an experimental structure of the target protein–bioactive molecule complex, an experimental structure of the apo protein can alternatively be used; ideally, this structure contains the appropriate subunit composition and was captured in the relevant functional state.
(3) When no experimental structure is available, homology modeling can generate a structural model of the target protein based on the experimental structure of a homologous template protein. When selecting the template structure, one should consider the sequence identity between the target and template and, additionally, other features of the template structure, such as the functional state and the bound ligand(s).
(4) Although the (experimental or computational) structure of the protein alone is already informative, carrying out a computational molecular docking of the bioactive molecule can help to further optimize the photoswitchable ligand design. In particular, the predicted binding mode can be used to identify the optimal position to introduce the photochromic group and/or estimate the length of the linker between the different modules of the PCL or PTL, as well as pinpoint potential residues for Cys screening.
(5) The photoswitchable ligand (PCL or PTL) design follows the modular approach depicted in Figure 1b. As mentioned in steps (1)–(4), structural information on the binding mode of the bioactive module to the target protein can be used to guide such a design.
(6) In the case of PTLs, their design additionally includes an inspection of the structure of the target protein, either experimental or computational, in order to identify putative tethering positions, i.e., residues near the ligand binding site amenable for cysteine mutagenesis screening.
(7) Upon design of the photoswitchable ligand, synthesis and experimental testing can already be performed; the latter includes measuring the modulatory effect of the ligand under different light conditions, as well as site-directed mutagenesis (either Cys mutations for PTL covalent attachment or other mutations to confirm the binding site location and PCL/PTL ligand binding mode).
(8) The observed light-dependent activity (or lack thereof), as well as the effect of mutations, can be rationalized *a posteriori* by performing a molecular docking of the PCL or virtual Cys mutation combined with covalent docking for the PTL. The resulting model of the target protein–photoswitch complex can be inspected to design additional site-directed mutations to validate the predicted PCL/PTL predicted binding mode. Alternatively, molecular docking can be used *a priori* (i.e., before experimental testing) to select the best candidate among several possible photoswitchable ligand designs (for subsequent experimental testing), as well as to explore alternative Cys tethering sites.
(9) Though the (static) computational models described so far are already useful to understand the molecular basis of light-controlled ion channel modulation, they can additionally be refined by molecular dynamics. Such simulations, alone or in combination with enhanced sampling and free energy techniques, can provide further dynamical and energetic insights into the photoswitch effect, as explained earlier in this section.
(10) This integrative computational-experimental approach offers a comprehensive understanding of the PCL/PTL effect on ion channel function, including but not limited to the information listed in the last step of the proposed workflow (see Figure 3).

In the following sections, we exemplify the steps of the proposed computational workflow (Figure 3) by discussing published computational modeling and simulation studies of photoswitchable ligands targeting VGICs and LGICs (see also Tables S1–S4).

## 3. Computational Modeling of Photoswitchable Ligands Targeting Voltage-Gated Ion Channels

**Figure 4:**
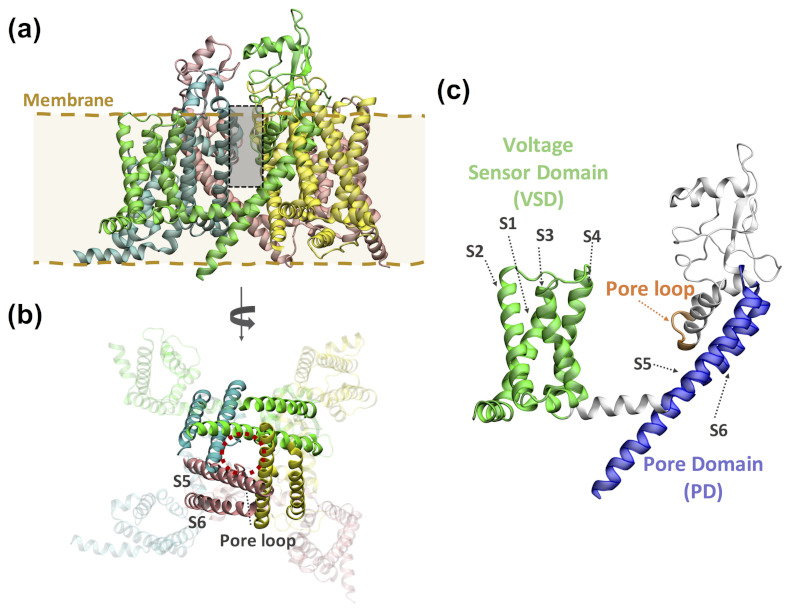
Representative structure of a tetrameric voltage-gated ion channel (VGIC). (**a**) Structure of the voltage-gated sodium channel Nav1.7, PDB code 6J8J [48], with protein subunits colored in green, yellow, pink and cyan, respectively. Only the pore-forming subunits are shown. The binding site of (photoswitchable) pore blockers is indicated with a gray box. (**b**) Alternative view of the same structure from the intracellular side. The helices displayed in solid colors correspond to the pore domain (S5 and S6 helices and the pore loop); the ion-conducting pore is indicated with a dashed red circle. (**c**) Detailed view of one Nav1.7 subunit indicating the two functional domains present in VGICs. The voltage sensor domain (VSD) has four transmembrane helices (S1–S4, colored in green) and the pore domain (PD) is constituted by two transmembrane helices (S5 and S6, in blue), connected by the pore (P-)loop, which contains the ion selectivity filter (in orange).

VGICs are membrane proteins whose ion conduction pores open and close in response to changes in membrane voltage, intracellular signaling molecules or both [45,46,47]. This superfamily contains voltage-gated potassium, sodium and calcium channels (Kv, Nav and Cav, respectively), and other members, such as the transient receptor potential (TRP) channels. Two functional domains can be present in this superfamily (Figure 4): the voltage sensing domain (VSD), constituted by four transmembrane helices (S1–S4), and the ion pore domain (PD), formed by two transmembrane helices S5–S6 connected by the pore (P-)loop, which contains the ion selectivity filter. Kv and TRP channels are tetrameric proteins in which each subunit contains a VSD and a PD, whereas in the Nav and Cav channels this tetrameric assembly is encoded in a single gene. Nonetheless, other members of the VGIC superfamily lack a PD (e.g., Kir, HV1 and TPTE channels) or are not tetrameric (such as K2P channels).

### 3.1. Photoswitchable Pore Blockers

A structural inspection of the first crystal structures of the KcsA potassium channel with TEA-like pore blockers [18,20] allowed the design of PTLs that target the homologous Shaker channel [21,22]. The ammonium binding site located in the extracellular vestibule of the pore (Figure 2b) appeared to be at the right distance from residue 422 (15–18 Å) to design a PTL (MAQ, **1** in Figure S1) whose quaternary ammonium group could reach this binding site when the azobenzene is in the *trans* configuration (approx. 17 Å long), but not in *cis* (~10 Å). The introduction of an E422C mutation for tethering and subsequent electrophysiological testing indeed revealed the photomodulation of the PTL-modified Shaker channel. Nonetheless, further structural information of the channel in complex with the photoswitchable ligand could help improve this initial design as well as characterize the molecular determinants of the differential effects of the *trans* and *cis* azobenzene forms.

In this regard, Mourot and coworkers [49] used a crystal structure of the Kv1.2–2.1 chimera in the open state and molecular docking to generate structural models of the Shaker K+ channel bound to a PCL composed of two quaternary ammonium moieties connected by an azobenzene group (QAQ, **2**). The extended shape of *trans*-QAQ places the two positively charged groups at the right distance to interact with the two quaternary ammonium binding sites inside the pore, one in the extracellular vestibule and the other below the selectivity filter. The latter cannot be occupied by *cis*-QAQ due to its bent shape, explaining why the *cis* form is a less potent pore blocker than the *trans* one. A similar computational approach was used to rationalize the functional effects of FHU-779 (**3**), a PCL composed of an azobenzene-based long tail and the Cav pore blocker diltiazem [50]. In this case, homology modeling was first used to generate a structural model of the Cav1.2 ion pore, based on an open-state structure of the bacterial NavAb sodium channel. Afterwards, molecular docking was performed by Monte Carlo-based minimizations. The computational models again showed that both isomers can be accommodated inside the pore. However, the elongated *trans*-FHU-779 extends along the pore, with the positively charged nitrogen near the selectivity filter, the adjacent benzothiazepine moiety bound to a lateral fenestration and the long photoswitchable tail interacting with the C-terminal region of helix S6. In contrast, the tail of the “folded” *cis* form cannot reach the latter region, explaining the reversible light-dependent block of Cav1.2 by FHU-779.

In order to get further molecular and energetic insights into the binding of pore blocker PCLs, a recent study [51] used MD simulations, together with an enhanced sampling technique (Gaussian accelerated MD) and free energy calculations (based on a molecular mechanics generalized Born surface area or MMGBSA approach). The VGIC studied was the Nav1.4 channel, for which a recent cryo-EM structure in the inactivated state is available, and *p*-diaminoazobenzene (**4**) was used as a simplified model of the aforementioned photoswitchable pore blockers. Interestingly, the simulations revealed that there is more than one binding site for *p*-diaminoazobenzene in the *trans* configuration. *p*-diaminoazobenzene binds to two binding sites compatible with its expected pore blocking activity, one in the central cavity near the selectivity filter and the other near the intracellular gate. In addition, the *trans* form of the PCL appears to bind in a lateral cavity close to the membrane, which includes some residues previously identified as important for the binding of local anesthetics. The occupancy of this third binding site suggests that *p*-diaminoazobenzene could act not only as pore blocker but also have similar effects to local anesthetics.

### 3.2. Photoswitchable Modulators

Photoswitchable ligands for VGICs are not limited to pore blockers and pore openers [52]. For instance, several PCLs have been reported for TRP channels that act as activators or inhibitors [53,54,55,56]. The structural information (either experimental structures or homology models) of TRP channels can then be used to understand the mechanism of ligand-mediated activation or inhibition. In this regard, Lichtenegger and coworkers [57] designed a photoswitchable analog of the endogenous activator diarachidonlyglycerol (OptoDArG, **5**) of the TRPC3 channel. A homology model of the TRPC3 channel was built (based on the cryo-EM structure of the closely related TRPV1) in order to design a mutagenesis screening of the lipid binding cavity. This screening revealed a single glycine residue that connects the binding pocket and the selectivity filter through a lateral fenestration, thus providing clues on how lipid sensing controls ion channel gating. To the best of our knowledge, molecular docking and MD simulations have not been applied yet to study the binding of photoswitchable lipids to TRP channels. However, these two computational techniques have been extensively used to investigate channel modulation by other TRP ligands [58,59,60,61,62] and thus their use could be easily extended to photopharmacological applications.

## 4. Computational Modeling of Photoswitchable Ligands Targeting Ligand-Gated Ion Channels

**Figure 5:**
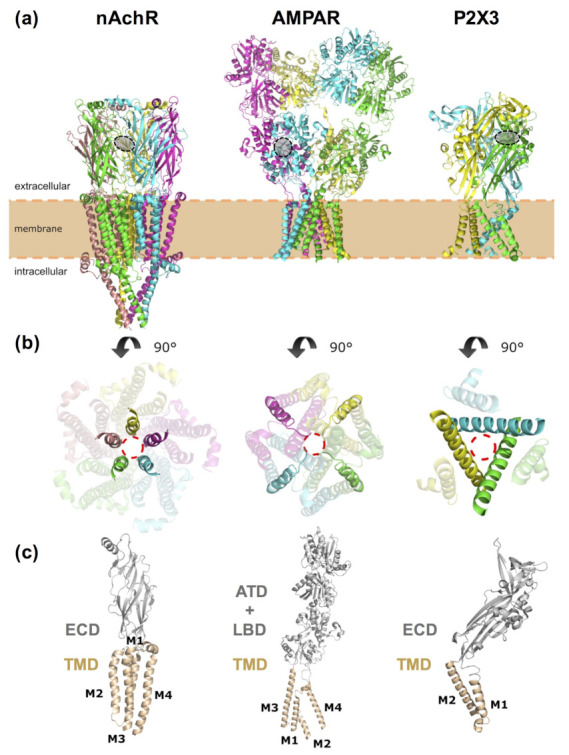
Representative structures of the three ligand-gated ion channel (LGIC) classes. (**a**) Shown from left to right, structures of a pentameric ligand-gated ion channel (pLGIC) based on the nicotinic acetylcholine receptor (nAchR), PDB code 7EKI [66]; an ionotropic glutamate receptor (iGluR), based on the AMPA receptor, PDB code 3KG2 [67]; and an ATP-gated purinergic (P2X) receptor, PDB code 5SVL [68]. Each protein subunit is shown in a different color (yellow, pink, green, cyan and/or magenta). A gray circle indicates the orthosteric binding site of LGICs, where the agonists or antagonists used to design most of the PCLs and PTLs mentioned in the text bind. For the sake of clarity, only one of the symmetric sites of the homomeric receptor is shown (out of the five present in nAchR, four in AMPAR and three in the P2X3 receptor). (**b**) Transmembrane domain (TMD) viewed from the extracellular side. The pore-lining structural elements are displayed in solid colors (i.e., the M2 helix for the nAchR and P2X3 receptors or the M2 helix and the re-entrant pore-loop for AMPAR) and the ion-conducting pore is indicated with a red dashed circle. (**c**) Detailed view of one subunit. The extracellular domains (ECD) of both the nAChR and P2X3 receptors are colored in gray, whereas the transmembrane domain (TMD) is colored in apricot. The AMPAR is displayed with the same color scheme, but the extracellular part of the receptor is divided into an amino terminal domain (ATD) and a ligand binding domain (LBD). Transmembrane helices are labeled from M1 to M4 or from M1 to M2, respectively. The intracellular domain is not shown for the sake of clarity.

LGICs are both ion channels that conduct ions across the neuronal membrane and receptors binding neurotransmitters. There are three main families [47,63,64]: pentameric LGICs (pLGICs), ionotropic glutamate receptors (iGluRs) and ATP-gated purinergic receptor (P2X) ion channels (Figure 5).

pLGICs [65] are composed of five identical or different subunits (homo- and hetero-pentamers, respectively). They encompass excitatory, cation-selective nicotinic acetylcholine receptors (nAchRs), serotonin or 5-hydroxytryptamine type 3 (5-HT${}_{3}$) receptors and zinc-activated channels (ZAC), as well as inhibitory, anion-selective γ-aminobutyric acid receptors (GABARs) and glycine receptors (GlyRs). In addition, prokaryotic members of the pLGIC family include the *Gloeobacter* ligand-gated ion channel (GLIC), a proton-gated cation-selective channel; the *Erwinia chrysanthemi* ligand-gated ion channel (ELIC), a cation-selective channel activated by small amines, such as GABA; and the *C. elegans* glutamate-gated chloride channel (GluCl). Each subunit of these pentameric receptors (Figure 5a) can be divided into an extracellular domain (ECD, contributing to the neurotransmitter binding site), a transmembrane domain (TMD, formed by four helices, M1-M4, of which M2 lines the ion conduction pore) and, in some cases, an intracellular domain (ICD).

iGluRs are LGICs essential for excitatory neurotransmission and can be classified in NMDA, AMPA and kainate receptors. They are tetrameric receptors (Figure 5b), with each subunit containing an extracellular amino terminal domain (ATD), an extracellular ligand binding domain (LBD, containing the glutamate binding site), a TMD (composed by three helices, M1, M3 and M4, as well as a re-entrant pore-loop, M2) and an intracellular carboxy-terminal domain (CTD).

Lastly, P2X receptors conduct mostly cations and are homo- or hetero-trimers (Figure 5c) composed of an ECD, a TMD (with two transmembrane helices, M1–M2, per subunit) and a C-terminal cytosolic tail.

### 4.1. Nicotinic Acetylcholine Receptors

nAchRs are the first ion channels for which light-modulated ligands were reported [11,12]. Based on a nAchR agonist, two photoswitchable ligands were designed [12]: bis-Q (**6** in  Figure S2), a PCL with two quaternary ammonium moieties linked by an azobenzene group, and QBr (**7**), a PTL composed of a quaternary ammonium, azobenzene and benzylic bromide for Cys tethering. Both bis-Q and QBr acted as agonists in the *trans* form, whereas the *cis* form was almost inactive. In contrast, azo-CarCh (**8**) and azo-PTA (**9**) were found to act instead as light-reversible antagonists [11], despite the fact that these two PCLs were also designed based on two known nAChR agonists (carbamylcholine and phenyltrimetylammonium, respectively). This strongly indicates that small changes in the photoswitchable ligand structure can dramatically affect its light-modulated activity and thus structural knowledge of the receptor is needed to improve the ligand design.

Structural information was used by Tochitsky and coworkers [69] to devise light-modulated nAchRs (LinAchRs) that can be activated or inhibited with light, while responding normally to acetylcholine. They used PTLs (MAAch and MAHoCh, **10** and **11** in Figure S2, respectively) consisting of a Cys reactive maleimide group, an azobenzene photoswitch and a ligand head group mimicking known nAChR agonists (acetylcholine and homocholine, respectively). Potential positions to introduce Cys mutations for PTL covalent attachment were identified by inspecting the experimental structure of the soluble AChBP (as a surrogate of the nAChR ECD) in complex with carbamylcholine (a known nAchR agonist) (Figure 2a), as well as a computational model of α4β2 nAchR in complex with MAAch (generated by combining homology modeling and molecular docking). The subsequent Cys screening showed that the E61C mutant was photomodulated by both PTLs, but with the opposite effects. Even though the design of both PTLs was based on nAChR agonists, E61C nAchR was photoactivated by *cis*-MAAch, but photoinhibited by *cis*-MAHoCh. The agonist activity of MAAch was further studied by repeating the aforementioned docking calculation with the homology model of α4β2 nAchR, but adding a positional constraint that restricted the maleimide group to be within a certain radius of the C61 sulfur atom. The obtained docking poses showed that only *cis*-MAAch, but not its *trans* form, can position the bioactive ligand headgroup in the right place. Although a similar calculation was not performed for MAHoCh, the unexpected antagonist activity of this PTL was rationalized based on structural information for other nAChR antagonists. A ‘foot-in-the-door’ mechanism was proposed [70], by which antagonist binding prevents the complete closure of the ligand binding site (in particular loop C), as required for receptor activation.

Molecular docking was also used to rationalize the differential effect of the *trans* and *cis* forms of a PCL targeting an insect nAchR [71]. AMI-10 (**12**) contains two molecules of imidacloprid, an AchR agonist normally used as an insecticide, linked by an azobenzene group. The PCL turned out to be more effective upon irradiation, with the *cis* form showing a median lethal dose fivefold lower than the *trans*. Using a structure of the sea slug AchBP as a surrogate of the insect nAchR, Xu and coworkers showed that *cis*-AMI-10 can place the two imidacloprid moieties inside the binding pocket, whereas for the *trans* form the second moiety extends outwards, without forming any interaction with the protein. The larger number of protein–ligand interactions for the *cis* isomer thus correlates with its higher insecticide activity. A recent study [72] has reported an alternative design, in which the two imidacloprid moieties are linked by a photoswitchable dithienylethene group (DitIMI) in order to improve solubility compared to azobenzene.

### 4.2. 5-HT_3_ Receptors

**Figure 6:**
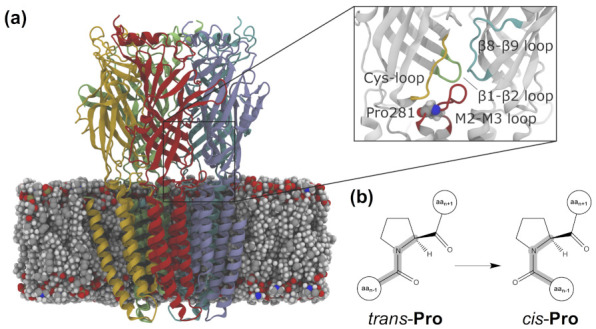
Prolyl isomerization in the serotonin or 5-hydroxytryptamine type 3 (5-HT${}_{3}$) receptor. (**a**) Receptor model embedded in a 1-palmitoyl-2-oleoyl-sn-glycero-3-phosphocholine (POPC) lipid bilayer. The five protein subunits are displayed in red, yellow, green, light blue, and blue, respectively, and the lipid molecules as van der Waals spheres. The inset shows the extracellular domain (ECD)–transmembrane domain (TMD) interface region where the isomerizable Pro281 (in van der Waals spheres) is located, surrounded by the Cys loop (in yellow), the β1–β2 loop (green), the β8–β9 loop (blue), and the M2–M3 loop (red). Adapted with permission from Crnjar, A.; Comitani, F.; Hester, W.; Molteni, C. Trans–cis proline switches in a pentameric ligand-gated ion channel: how they are affected by and how they affect the biomolecular environment. *J. Phys. Chem. Lett.* **2019**, *10(3)*, 694–700. Copyright 2019 American Chemical Society. (**b**) Schematic representation of the *trans-cis* isomerization of proline.

The 5-HT${}_{3}$ receptor is another important cationic pLGIC, activated by serotonin, which is involved in a series of neurological disorders, from schizophrenia to drug abuse. Pharmacologically, it is the target of several drugs, including antiemetics, which act as antagonists, to alleviate the effects of cancer therapies [73]. To the best of our knowledge, only one study has explored and experimentally characterized azologs of reported antagonists of the 5-HT${}_{3\text{A}}$ receptor [74], with only one of the investigated compounds retaining antagonist activity with no isomer specificity. Complementarily *trans-cis* switches based on Pro analogs have been used to study the gating mechanism of the 5-HT${}_{3}$R. Although still a controversial mechanism, it has been proposed that the *trans*-to-*cis* isomerization of a Pro residue located in the loop connecting the M2 and M3 helices at the ECD–TMD interface (Pro8* or Pro281 in the X-ray structure of the mouse 5-HT${}_{3}$R [75]) mediates channel gating. Mutations of this Pro into unnatural amino acid analogs strongly favoring the *trans* isomer resulted in non-functional channels [76], suggesting that, if *trans-cis* isomerization of Pro8* cannot occur, the channel would not open. A follow-up computational study [77] used MD simulations combined with enhanced sampling (metadynamics) to investigate the isomerization of a series of proline analogs (i.e., the ones tested in the aforementioned mutagenesis experiments) using dipeptide model systems in aqueous solution. A comparison of these simulations with the electrophysiology data showed an excellent correlation between the calculated free energy differences between the *cis* and *trans* isomers and the effect of the unnatural mutations on the receptor functional response. However, these simulations were performed on simplified models and thus did not take into account the effects of the receptor environment. These were addressed in subsequent molecular dynamics and metadynamics simulations of a model of the 5-HT${}_{3}$R, built based on an X-ray structure [75] including the extracellular and transmembrane domains, embedded in a 1-palmitoyl-2-oleoyl-sn-glycero-3-phosphocholine (POPC) lipid bilayer (Figure 6a). These simulations showed how the protein environment affects the proline isomerization free energy landscape, which loses symmetry with respect to the case in water [78]. In addition, they provided the molecular details of the network of interactions of the proline potential switch with other residues at the ECD–TMD interface. On the one hand, such interactions select a preferential isomerization path. On the other, Pro isomerization causes the constriction of a ring of negatively charged Asp residues at the top of the pore-lining helix, which might enhance cation attraction and conduction. Altogether, the Pro molecular switch (Figure 6b) appears to behave as the endogenous counterpart of photoswitchable ligands (e.g., Glyght; see Section 4.4). In addition to 5-HT${}_{3}$R, other ion channels [79] seem to control activation by using prolyl isomerization, which is additionally involved in many other biological processes [80,81]. Complementarily, the recent development of light-sensitive unnatural amino acids (UAAs) [82] has opened the way to endow light sensitivity to ion channels directly using these UAA probes.

### 4.3. GABA_A_ Receptors

GABA${}_{\text{A}}$ receptors are highly pharmacologically relevant pLGICs [83,84,85], being the targets of benzodiazepines for the treatment of anxiety, insomnia and depression, as well as of clinically used anesthetics (such as etomidate and propofol). The rich pharmacology of GABA${}_{\text{A}}$ receptors offers a wide variety of starting options for the design of photoswitchable ligands. Nonetheless, until recently, such efforts were hampered by the lack of experimental structural information on the ligand binding sites [86,87,88]. The design of photoswitchable ligands would then rely on ligand structure–activity relationship data, as well as mutagenesis and Cys scanning data for the receptor binding sites. PCLs and PTLs were thus generated, targeting the orthosteric (GABA) binding site [89,90,91] or allosteric binding sites [92,93,94].

Homology modeling and molecular docking have been used to further characterize the experimentally observed photomodulation at the molecular level. For instance, Lin and coworkers [89] used a homology model of the receptor to map the potential tethering sites for Cys mutation in the orthosteric site and thereby design a light-modulated GABA${}_{\text{A}}$R (LiGABA${}_{\text{A}}$R), formed by α1(T125C), β2 and γ2S subunits. This model was built based on the experimental structures of AchBP (as a surrogate for the ECD of pLGICs) and the related *Torpedo* nAChR (for the TMD) [95]. Furthermore, molecular docking was performed to rationalize the antagonist effect of one of the proposed PTLs, MAB-0 (**13** in Figure S3), composed of maleimide, azobenzene and 4-hydroxybenzylamine. Although the latter group is not a typical gabaergic agonist/antagonist, in the *trans* form it appears to interact with several aromatic residues in the orthosteric site, thus enabling competitive inhibition against GABA. Instead, the *cis* isomer is not able to place the 4-hydroxybenzylamine near the putative interacting residues, consistent with its lack of effect on channel function. Homology modeling and molecular docking were also used in a recent study [96] to rationalize the different binding preferences of azogabazine (**14**). This PCL is composed of azobenzene and gabazine, a known GABA${}_{\text{A}}$R competitive antagonist binding to the orthosteric site. In this case, the homology modeling approach benefited from the resolution of the first cryo-EM structures of heteropentameric GABA${}_{\text{A}}$Rs. The model for the murine α1β2γ2L receptor was built using the cryo-EM structure of human α1β3γ2L GABA${}_{\text{A}}$R as a template [97]. The *trans*-azogabazine docking poses revealed protein–ligand interactions similar to those observed in cryo-EM structures of other GABA${}_{\text{A}}$R–antagonist complexes, explaining its antagonist activity. In addition, the *trans*-azogabazine docking poses were used to design site-directed mutagenesis experiments, which further confirmed the predicted binding mode [96].

The computational methods described above have also been used to investigate the binding of photoswitchable ligands to allosteric sites. Borghese and coworkers [98] used docking to identify possible tethering points in a α1β3γ2 GABA${}_{\text{A}}$R for MAP20 (**15**), a PTL composed of methanethiosulfonate, azobenzene and the anesthetic propofol. Capitalizing on a previously published model [99,100] of the parent compound propofol bound to the TMD binding site at the β+α- interface [101], they manually superimposed the anesthetic part of the PTL and then sampled the rotations of the bond connecting propofol to azobenzene to identify nearby receptor residues amenable for Cys mutation. Experimental testing of the proposed Cys mutants showed that β3(M283C) and α1(V227C) are the two Cys mutants displaying the largest photomodulation of GABA-induced currents upon treatment with MAP20. Subsequent structural modeling of MAP20 conjugated at these positions showed that the PTL can reach one or two propofol β+α- binding sites depending on the tethering subunit (α1(V227C) or β3(M283C), respectively). The predicted number of occupied binding sites is in line with the photomodulation of α1β3(M283C)γ2 GABA${}_{\text{A}}$R being larger than for α1(V227C)β3γ2. Moreover, the computational models were validated retrospectively by comparison with a cryo-EM structure of GABA${}_{\text{A}}$R bound to propofol [102].

**Figure 7:**
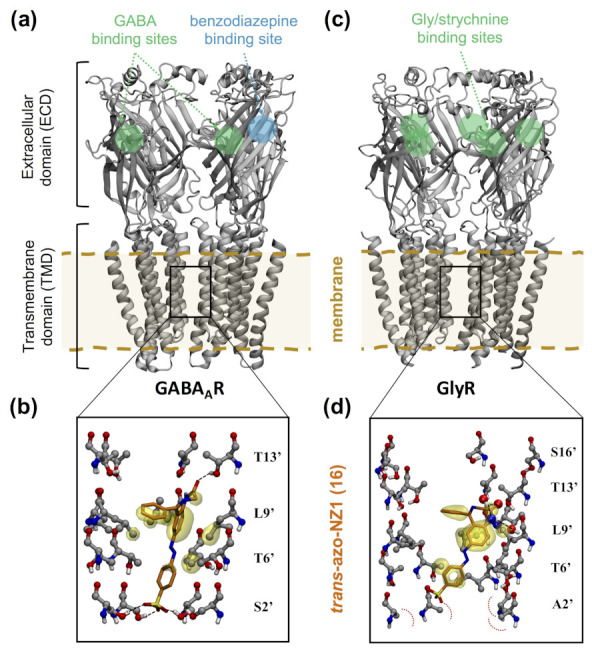
Computational models of the pore blocker *trans*-azo-NZ1 bound to γ-aminobutyric acid type A (GABA${}_{\text{A}}$) and Gly receptors. (**a**) Computational model of α1β2γ2 GABA${}_{\text{A}}$R in complex with GABA [105]. The orthosteric GABA binding sites and the allosteric benzodiazepine binding site in the ECD are indicated with green and blue circles, respectively. For the sake of clarity, the front subunit of the heteropentamer is not shown. (**b**) Docking pose of *trans*-azo-NZ1 in the GABA${}_{\text{A}}$R-ρ2 pore. Pore-lining residues are labeled according to the generalized numbering of the M2 helix residues for pLGICs. Hydrophobic interactions between azo-NZ1 and the receptor residues are marked as yellow transparent surfaces, hydrogen bond interactions and steric repulsion are represented with black and red dashed lines, respectively. Adapted from reference [103] with permission (CC-BY NC license) from the British Journal of Pharmacology, published by John Wiley and Sons (2019). (**c**) Cryo-EM structure of GlyR in complex with strychnine [109], a competitive antagonist that binds to the glycine neurotransmitter site, whose location is indicated with a green circle. For the sake of clarity, the front subunit is not shown. (**d**) Docking pose of *trans*-azo-NZ1 in the G2’A α1 GlyR pore. Interactions are displayed following the same representation as in panel (**b**). Adapted from reference [110] with permission (CC-BY license) from eNeuro, published by the Society for Neuroscience (2020).

Homology modeling and molecular docking have been used to rationalize the unforeseen photomodulatory effects of azo-NZ1 (**16**) [103]. This PCL was based on the benzodiazepine nitrazepam, which was conjugated with the azobenzene photoswitch and additionally a sulfonate group to improve solubility. Thus, the PCL design aimed at creating a photoswitchable positive allosteric modulator binding at the benzodiazepine binding site of GABA${}_{\text{A}}$R, located at the α+γ- interface in the ECD. Instead, electrophysiological data showed that *trans*-azo-NZ1 acts as a GABA${}_{\text{A}}$R blocker, i.e., binds inside the TMD pore. Moreover, *trans*-azo-NZ1 inhibited GABA-mediated currents for some GABA${}_{\text{A}}$R-ρ (or GABA${}_{C}$R [104]) subtypes, as well as Gly-mediated currents for some GlyRs. This was completely unexpected, since GABA ρ subunits and GlyRs are insensitive to benzodiazepines. In order to understand the complete change of pharmacological activity of azo-NZ1 compared to the parent benzodiazepine, electrophysiological and mutagenesis experiments were combined with computational modeling. The homology model of α1β2γ2 GABA${}_{\text{A}}$R was taken from reference [105] (Figure 7a), whereas the homology models of GABA${}_{\text{A}}$R-ρ were built following reference [106]. The main templates for these models are crystal structures of the homologous glutamate-gated chloride channel GluCl in the open state [107] and thus the resulting models are in the right functional state to study pore blockade upon GABA-triggered opening. Molecular docking (with flexible side chains for the pore-lining residues) showed that *trans*-azo-NZ1 has the right length to extend along the TMD ion pore (Figure 7b). The benzodiazepine core is positioned in the upper part of the TMD pore, forming hydrogen bonds with the 13’ residues (following the generalized numbering of the M2 helix residues for pLGICs) and hydrophobic interactions with the 6’ and 9’ residues, whereas the sulfonate group binds at the lower part (near the 2’ position). Interestingly, the negatively charged sulfonate overlaps with one of the chloride ion binding sites detected in cryo-EM structures [108]. Therefore, *trans*-azo-NZ1 hampers chloride conduction both sterically and electrostatically, consistent with the experimentally observed inhibitory effect. In contrast, the *cis* isomer binds in the middle region of the pore, which is wider, and thus blockade is less likely, in line with the inhibition relief upon UV irradiation. The docking calculations also provided molecular insights into the subtype selectivity of azo-NZ1 [103]. Electrophysiological experiments showed that both the heteropentameric α1β2γ2 GABA${}_{\text{A}}$R and the homopentameric GABA${}_{\text{A}}$R-ρ2 were inhibited by *trans*-azo-NZ1, whereas GABA${}_{\text{A}}$R-ρ1 was not. Moreover, sensitivity to azo-NZ1 depended on the residue at position 2’: the S2’G GABA${}_{\text{A}}$R-ρ2 mutation abolishes inhibition, whereas the P2’S GABA${}_{\text{A}}$R-ρ1 mutation endows sensitivity to azo-NZ1. Based on the docking results on the three GABA${}_{\text{A}}$Rs [103], it was proposed that *trans*-azo-NZ1-mediated inhibition requires either a hydrogen-bonding Ser or a residue of similar volume (Ala or Val) at position 2’.

### 4.4. Glycine Receptors

Compared to GABA${}_{\text{A}}$Rs, the number of (photo)pharmacological agents for GlyRs is more limited [111,112]. Nonetheless, GlyRs are attracting growing attention as possible targets for painkillers [112,113]. Two PCLs based on azobenzene and a benzodiazepine core have been developed so far [110,114]. The aforementioned azo-NZ1 (**16** in Figure S3), as well as Glyght (**17**), exhibited light-modulated responses in an in vivo behavioral zebrafish assay. In vitro receptor screening using an heterologous expression system revealed that *trans*-azo-NZ1 acts as pore blocker for both GABA${}_{\text{A}}$Rs and GlyRs, whereas *cis*-Glyght is a GlyR-selective negative allosteric modulator.

The *trans*-azo-NZ1-mediated pore blockade across several pLGICs resembles the inhibition mechanism of other pore blockers, such as picrotoxin [115,116]. Similar to GABA${}_{\text{A}}$Rs [103], homology modeling and molecular docking were used to provide molecular insights into the binding of azo-NZ1 inside the GlyR ion pore, as well as to explain the GlyR subtype selectivity [110]. Capitalizing on the availability of cryo-EM structures of homomeric α1 GlyR [109] (Figure 7c), models of the homopentameric G2’A α1 GlyR mutant (as surrogate of α2 GlyR) and the heteropentameric α2/β GlyR were built. The subsequent docking calculations showed that the binding mode of azo-NZ1 inside the GlyR ion pore is very similar to the one of GABA${}_{\text{A}}$Rs, with the elongated *trans* isomer extending from the 13’ to the 2’ position (Figure 7d). The sulfonate group is still accommodated at the 2’ position, despite the lack of a hydrogen-bonding residue, because the volume of the Ala 2’ residue (present in GlyRs containing wild-type α2 and G2’A α1 mutant subunits) is similar to that of the Ser residue at the same position in ρ2 and γ2 GABA${}_{\text{A}}$R subunits. Instead, the Gly 2’ residue in GlyRs containing wild-type α1 subunits is smaller; as a result, *trans*-azo-NZ1 is not able to completely block the pore, in line with the reduced inhibitory effect of the PCL for this GlyR subtype.

**Figure 8:**
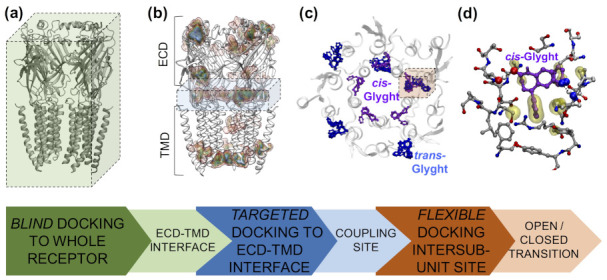
Multilevel docking screening to identify and characterize the binding site of Glyght in GlyR. (**a**) Blind docking of Glyght to the cryo-EM structure of α1 GlyR [109]. For the sake of clarity, the front subunit is not shown. The box size used for the blind docking calculation, encompassing the whole receptor, is shown as a green box. (**b**) Density map of the ligand poses of *cis*-Glyght obtained in the blind docking. Each contour line corresponds to a number density of 0.0006 particles/Å^3^. Among the several high-density regions, the interface between ECD and TMD appears to be the most likely binding region for *cis*-Glyght [114], as it shows the largest differences compared to the *trans*-Glyght blind docking (data not shown). The box size for the subsequent targeted rigid docking is indicated as a blue box. (**c**) Docking poses obtained by rigid docking focused on the ECD–TMD interface. The five α1 GlyR subunits are represented in ribbons and colored in white, whereas the most populated ligand poses of *cis*- (violet) and *trans*-Glyght (blue) are shown as ball and sticks. The box size for the subsequent flexible docking is indicated as an orange box. (**d**) Detailed view of the *cis*-Glyght binding pose obtained by flexible docking centered at the ECD–TMD interface site. Hydrophobic contacts between *cis*-Glyght and the receptor residues are represented as a yellow surface, while the atoms involved in receptor–ligand hydrogen bonds are represented with larger spheres and a dashed line between them. Adapted from reference [114] with permission from Cell Chemical Biology, published by Elsevier (2020).

As mentioned above, the selective inhibitory effect of Glyght (**17**) on GlyRs when in *cis* form [114] was completely unexpected. The PCL was designed based on a nitrazepam/diazepam core and GlyRs do not contain a benzodiazepine binding site. Hence, a multilevel screening approach (Figure 8) was used to uncover the possible binding site of Glyght. A blind docking calculation was first run to identify putative binding pockets on the surface of the complete receptor. The results indicated that Glyght might potentially bind at the interface between ECD and TMD (Figure 8a,b). Therefore, an additional docking calculation was run, focused on this region. *Cis*-Glyght was found to bind in five symmetric sites located between two adjacent subunits in the homopentameric structure (Figure 8b,c). This region has been shown to participate in the allosteric coupling between neurotransmitter binding in the ECD and opening of the TMD ion pore [64]. Thus, it is likely that ligand binding at the ECD–TMD interface can thereby interfere in receptor activation. Lastly, the Glyght docking poses were further refined by using flexible docking centered on one out of the identified five symmetric intersubunit sites (Figure 8b,c). *Cis*-Glyght strengthens the interaction between M2–M3 and β8–β9 loops, which stabilizes the closed state. Such a “stapling” mechanism is in agreement with the stronger GlyR inhibition by *cis*-Glyght observed experimentally. Interestingly, the Glyght binding site in GlyR overlaps with the ECD/TMD interface region where the Pro molecular switch in 5-HT${}_{3}$ receptors is located [76,77,78]. Thus, the light-modulated effect of Glyght on GlyR resembles the mechanism by which *trans-cis* Pro isomerization may mediate channel gating in the 5-HT${}_{3}$ receptor [76,77,78]. This further supports the idea of the Pro molecular switch behaving as the endogenous counterpart of photoswitchable ligands.

### 4.5. Ionotropic Glutamate Receptors

The design of the first photoswitchable ligands targeting ionotropic glutamate receptors [24,25,28] was based on inspection of X-ray structures of the LBD of the kainate receptor (GluK2, formerly known as GluR6) in complex with different ligands [23]. The LBD has a clamshell-like structure and the degree of closure upon ligand binding correlates with the degree of receptor activation. Moreover, these structures revealed an “exit tunnel” between the lips of the clamshell that could potentially accommodate the elongated *trans* form of the azobenzene photochrome. Based on these structural data, a PCL was designed consisting of the glutamate agonist and an azobenzene photoswitch (4-GluAzo, **18** in Figure S4), which acted as an agonist in the *trans* form but was inactive in the *cis* form [24], as intended with the original structure-guided design. In 2013, the crystal structure of GluK2 in complex with 4-GluAzo was solved [19] and confirmed the predicted binding mode of the *trans* isomer (Figure 2c). The glutamate group of 4-GluAzo interacts similarly to the endogenous glutamate agonist, while the *trans*-azobenzene is positioned between the lips of the clamshell. Complementarily, a manual docking calculation of the *cis* isomer on the same X-ray structure indicated that the distal phenyl ring in the *cis* configuration would clash with the lips of the clamshell and thus cause the opening of the LBD, explaining why 4-GluAzo is inactive upon irradiation. A follow-up computational study [117] used MD simulations together with umbrella sampling-based free energy calculations to further characterize the structural rearrangements occurring in the LBD upon PCL isomerization, as well as to estimate the change in binding affinity between *trans*- and *cis*-4-GluAzo.

An analysis of the available GluK2 X-ray structures [23] also allowed the design of a Cys mutagenesis screening to identify possible tethering sites of PTLs. Thereby, a light-modulated ionotropic glutamate receptor (LiGluR) was developed, containing a L439C mutation [25], whose activity was modulated by MAG compounds. Such PTLs consisted of maleimide, azobenzene and the agonist glutamate; the different modules were linked by one or more glycine units in order to vary the tether length. The first MAG compounds reported acted as photoswitchable LiGluR agonists when in the *cis* form [24,25,28]. Nonetheless, a subsequent study [118] reported other MAG compounds that were active in either the *cis* or *trans* forms, depending on the PTL tether length and the position where the tethering Cys was introduced (L439C or G486C). A model of the GluK2 LBD bound to the PTL MAG0 (**19**) was built based on the X-ray structure of the protein in complex with the parent compound glutamate [23]. Subsequent MD simulations and umbrella sampling-based free energy calculations [118] revealed that two factors contribute to determine which photoisomer is active: (i) the probability to properly orient the glutamate moiety inside the binding site of the open LBD and (ii) the degree of clamshell closure upon ligand binding.

A similar structure-based strategy was used to design photoswitchable ligands for other ionotropic glutamate receptors. In the case of NMDARs, light-modulated receptors (LiGluNs) were designed based on a structure-guided Cys mutagenesis screening to identify possible tethering sites for PTLs of the MAG series [119]. As described above for MAG compounds and LiGluRs, the attachment point and the length of the linker can yield either LiGluNs that are photoactivated (LiGluN2A-V173C or LiGluN2B-V714C) or photoinhibited (LiGluN1A-G172C and LiGluN1a-E406C) by MAGs. In the case of AMPAR, the crystal structure of the GluA2 LBD in complex with a benzyltetrazolyl-substituted AMPA (BnTetAMPA) [67] showed that the clamshell topology of the LBD is conserved among iGluRs. However, the degree of closure upon ligand binding is tighter for AMPARs compared to kainate receptors. As a result, the benzyl substituent of the AMPA analog is located in a cleft that opens into the solvent, suggesting that, upon replacement with azobenzene, the photochrome could still be accommodated in this cleft, but only if the azo group is added in the meta (or 3) position with respect to TetAMPA [120]. Hence, a new series of PCL compounds targeting AMPAR was designed called ATA; said compounds are composed of the azobenzene photoswitch, a tetrazolyl linker and the AMPA agonist [120]. The ATA PTLs were active in the *trans* form, as intended, and were selective for AMPARs over kainate receptors. A follow-up study [121] combined flexible docking, MD simulations and umbrella sampling-based free energy calculations to rationalize the differential effect of the two isomers of one of such ATA compounds, ATA-3 (**20**). Two ligand binding modes were found. One is similar to the crystallographic poses of other AMPAR agonists and corresponds to the most stable pose for the active *trans* isomer. The other could represent the position of the ligand immediately upon photoisomerization, in which a hydrogen bond is lost. From this second binding mode, the *cis* isomer could easily dissociate, unless it switches back to *trans*.

Photoswitchable ligands targeting ionotropic glutamate receptors are not limited to PCLs and PTLs binding to the LBD. A recent study [122] reported a photoswitchable pore blocker for the glutamate delta2 (GluD2) receptor. Glutamate delta receptors belong to the iGluR family due to their sequence and structure similarity to AMPA, NMDA and kainate receptors. However, they are considered to be orphan receptors because their LBD does not bind glutamate; instead, pore opening is regulated indirectly by metabotropic G protein-coupled glutamate receptors. Hence, Lemoine and coworkers [122] devised a PTL based on a known pore blocker (pentamidine), instead of an agonist binding to the LBD. MAGu (**21**) is composed of maleimide, azobenzene and a guanidinium head group that mimics the positively charged groups of pentamidine. The *trans* isomer is expected to reach the inside of the pore due to its elongated shape, whereas the shorter *cis* isomer will not. In order to identify possible positions to covalently attach MAGu on the pore-lining M3 helix, a homology model of GluD2 was built based on a crystal structure of the GluA2 receptor in the activated state. The thus-designed I677C mutant is blocked by *trans*-MAGu, but not by the *cis* form, and is denoted as the light-controllable GluD2 (LiGluD2) channel. However, differently to pentamidine, the blockade by MAGu was not dependent on membrane voltage, suggesting that the two molecules may have different binding sites. This prediction was tested by performing covalent docking and ion pore calculations [122]. The positively charged group of MAGu does not seem to reach the inside of the pore, in line with the different blocking properties of MAGu and pentamidine observed experimentally. Instead, MAGu appears to alter the geometry and electrostatics of the pore, thus affecting ion conduction.

### 4.6. P2X Receptors

A recent review has compiled the photopharmacology applications for purinergic receptors reported until June 2021 [123]. Here, we showcase a study combining the use of a photoswitchable ligand and molecular dynamics simulations [124]. The used PTL (MAM, **22**) contains two maleimide groups separated by an azobenzene photoswitch, so that end-to-end distance of this Cys-Cys crosslinker changes with light. As previously carried out for other PTLs [125], the Cys screening of the transmembrane helices was designed using two homology models of the P2X2 receptor, based on experimental structures of the homologous P2X4 receptor in either closed (apo) or open (ATP-bound) states [126,127]. According to the experimental data for the P2X2 receptor, the two most promising Cys mutations were introduced into the equivalent positions (I336C and N353C) of the two aforementioned structures of the P2X4 receptor. Then, the MAM molecule was attached to these Cys mutants in either a “horizontal” (I336C/I336C mutant) or a “vertical” (I336C/N353C mutant) configuration and MD simulations were run for both P2X2 states. The simulations provided structural insights supporting the ideas that the P2X pore fluctuates among different open conformations and that receptor activation involves bending of the M2 helices at a conserved glycine residue that acts as a hinge for gating.

## 5. Conclusions and Perspectives

Light-controlled modulation of ion channels can be achieved by using photoswitchable ligands whose two isomers display different binding properties. Unraveling the molecular details of the complex between the target protein and the photoswitchable ligand in each of its two forms can help optimize this differential effect. Moreover, the addition of the photochromic group can modify the pharmacological properties of the parent bioactive molecule. Furthermore, the covalent attachment position and the length of the linker of photoswitchable tethered ligands can also result in different light-modulated effects. Given this complexity, a rational structure-based design approach is strongly recommended [2,6,28].

Indeed, the increasing availability of X-ray and cryo-EM structures has accelerated the development of both PCLs and PTLs for both VGICs [21,22] and LGICs [24,25]. Such experimental structural information has been complemented by computational modeling, in particular, homology modeling, molecular docking and molecular dynamics (Figure 3). In addition to providing structural models for ion channels without available experimental structures, these computational methods allow the modeling of the target protein bound to the photoswitchable ligand in either of its two forms. A comparison of the two counterpart models can be carried out either *a posteriori* (to explain the molecular basis of the observed light-modulation) or *a priori* (to predict whether the two photoswitch isomers will bind differently, as intended during the design). In the case of PTLs, covalent attachment can also be included in the model and thereby the fitness of the Cys mutant and the linker length can be explored for positioning the bioactive group in the correct binding pocket. Hence, nowadays, many photopharmacology studies integrate computational structural modeling together with the experimental data (Tables S1–S4). Based on the successful results obtained so far, we expect that computational methods will become instrumental in the field of ion channel photopharmacology in the coming years, especially when considering the ongoing methodological developments in the field.

Recent advancements in membrane protein structural biology, in particular cryo-EM, have substantially increased the number of available ion channel and receptor structures [128,129] that can be used as input to design photoswitchable ligands. In this regard, a recent study has reported crystal structures of the glutamate transporter homologue Glt_Tk_ in complex with a photoswitchable inhibitor in either *cis* or *trans* form [129]. In addition, these new experimental structures have broadened the range of templates available to build homology models for other ion channels without experimental structures. Furthermore, computational structural models can now be generated not only through homology modeling, but also using recently developed machine learning-based methods, such as AlphaFold [130] and RoseTTaFold [131].

Together with these computational protein structures, the accuracy of the predicted photoswitchable ligand binding modes is expected to improve thanks to the continuous development of molecular docking techniques and scoring functions [132]. Covalent docking methods [133,134] will be particularly useful for PTLs, whereas quantum mechanics (QM)-based docking approaches [135,136] may be applied to both PCLs and PTLs.

Complementarily, it is expected that the steady increase in computational resources, as well as the development of more efficient MD algorithms and enhanced sampling/free energy techniques [137,138,139,140], will also pave the way for further studies using classical simulations of photoswitchable ligands in complex with their target proteins. However, special care will be needed to develop accurate and transferable force field parameters for the two photoswitch forms [141]. Quantum mechanics/molecular mechanics (QM/MM) MD [142], in combination with excited state methods, will also allow us to study the photoisomerization of the azobenzene group within the PCL or PTL, either in solution or bound to the target protein. Nonetheless, going from the previous studies of azobenzene in solution [143,144] to photoswitchable ligands in complex with their target protein is likely to require adjustments in the theoretical treatment of the photochromic group excited states, as well as to address possible super-heating effects due to photoexcitation.

Hand in hand with these computational advancements, the toolkit of available photoswitchable ligands is rapidly expanding. The development of photoswitchable amino acids [82] and lipids [145] opens new avenues to investigate ion channel regulatory mechanisms. MD simulations are expected to be particularly useful to characterize at the molecular level the effect of these novel photoswitchable molecules [42,78,146,147]. Although the PCLs and PTLs mentioned in this review are based on azobenzene and its *trans-cis* isomerization upon light irradiation, other photochromic groups are increasingly being used, such as fulgimides [148], diarylethenes [149] and stilbenes [150], for which photoswitching involves bond formation. In these cases, the aforementioned QM- and QM/MM-based approaches could be combined with docking, MD and/or excited state calculations, to model the light-induced bond formation, providing an unprecedented atomistic picture of these exciting photoswitching processes.

## Supplementary Materials

The following are available online at https://www.mdpi.com/article/10.3390/ijms222112072/s1.
