# Supplementary material for: Photopharmacology of Ion Channels through the Light of the Computational Microscope
Source: Int J Mol Sci. 2021 Nov 8;22(21):12072. doi: 10.3390/ijms222112072 (PMC8584574; doi:10.3390/ijms222112072)
Supplement: Supplementary file 1 [file ijms-22-12072-s001.zip › ijms-1436354-supplementary.pdf]

Photopharmacology of ion channels through the light of the computational microscope

Supplementary Material

Alba Nin-Hill[1], Nicolas P. Mueller[2,3], Carla Molteni[4], Carme Rovira[1,5] and Mercedes Alfonso-Prieto [2,6,*]

[1] Departament de Química Inorgànica i Orgànica (Secció de Química Orgànica) and Institut de Química Teòrica i Computacional (IQTCUB), Universitat de Barcelona, 08028 Barcelona (Spain)
[2] Institute for Advanced Simulations IAS-5 and Institute of Neuroscience and Medicine INM-9, Computational Biomedicine, Forschungszentrum Jülich, Jülich 52425 (Germany)
[3] Faculty of Mathematics and Natural Sciences, Heinrich-Heine-University Düsseldorf, Universitätsstr.1, 40225 Düsseldorf (Germany)
[4] Physics Department, King's College London, Strand, London WC2R 2LS (U.K.)
[5] Institució Catalana de Recerca i Estudis Avançats (ICREA), 08020 Barcelona (Spain)
[6] Cécile and Oskar Vogt Institute for Brain Research, University Hospital Düsseldorf, Medical Faculty, Heinrich Heine University Düsseldorf, Düsseldorf 40225 (Germany)

* Correspondence: m.alfonso-prieto@fz-juelich.de

Table S1. Computational studies of photoswitchable ligands targeting voltage-gated ion channels. Both photochromic ligands (PCLs) and photoswitchable tethered ligands (PTLs) are included.

Target protein	Tethering mutations	Photoswitchable ligand	Type	Activity	Computational methods	Aim	Reference
Shaker Kv channel	n.a.	QAQ (quaternary ammonium + azobenzene + quaternary ammonium)	PCL[i]	Pore blocker (trans)	Molecular docking	Rationalization of differential effect of trans and cis isomers	[1]
Cav1.2 channel	n.a.	FHU-779 (azobenzene + diltiazem)	PCL	Pore blocker	Homology modeling + Molecular docking (Montecarlo minimization)	Rationalization of differential effect of trans and cis isomers	[2]
Nav1.4 channel	n.a.	azobenzene & p-diamino-azobenzene	PCL[ii]	Pore blocker (trans)	Molecular dynamics + Free energy calculations (GaMD + MM/PBSA)	Understanding the mechanism of pore blocker binding	[3]
TRPC3 channel	n.a.	OptoDArG (azobenzene-containing-arachidony l+ glycerol)	PCL	Activator	Homology modeling	Structure-guided mutagenesis screening to understand the lipid-sensing and gating mechanism	[4]

[i] QAQ acts as light-regulated open channel blocker not only for voltage-gated K^+ channels, but also voltage-gated Na^+ and Ca^{2+} channels [1].
[ii] Azobenzene and p-diaminoazobenzene were used as simplified models of the light-sensitive pore blocker QAQ [3].

Table S2. Computational studies of photoswitchable ligands targeting cationic pentameric ligand-gated ion channels.

Target protein	Tethering mutations	Photoswitchable ligand	Type	Activity	Computational methods	Aim	Reference
nAchR (α4β2)	LinAChR (β2(E61C))	MAACh (maleimide + azobenzene + acylcholine)	PTL[i]	Agonist (cis)	Homology modeling+ Molecular docking	Structure-guided mutagenesis screening to identify possible tethering sites (Cys mutants) & Rationalization of differential effect of trans and cis isomers	[5]
nAchR	n.a.	AMI-10 (azobenzene + bis- imidacloprid)	PCL[ii]	Agonist (cis)	Molecular docking	Rationalization of differential effect of trans and cis isomers	[6]
5-HT3R	n.a.	Pro8*	n.a.[iii]	Endogenous molecular switch	Molecular dynamics + Free energy calculations (metadynamics)	Molecular and energetic insight into the connection between prolyl isomerization and channel gating	[7,8]

[i] Modeling of MAHoCh (maleimide+ azobenzene+ homocholine), another PTL reported in reference [5], was not attempted, due to the lack of ligand similarity between this PTL and the antagonists for which crystal structures bound to AChBP were available at the time.
[ii] The AchBP was used as proxy of the target nAchR in flies.
[iii] Although the endogenous Pro molecular switch of 5HT3R is not a photoswitchable ligand, it also undergoes *trans-cis* isomerization resulting in changes in channel activity.

Table S3. Computational studies of photoswitchable ligands targeting anionic pentameric ligand-gated ion channels.

**Target protein**	**Tethering mutations**	**Photoswitchable ligand**	**Type**	**Activity**	**Computational methods**	**Aim**	**Reference**
$GABA_AR$ (α1β2γ2S)	$LiGABA_AR$ (α1(T125C))	MAB-0 (maleimide+ azobenzene+ 4-hydroxy-benzylamine)	PTL	Antagonist (trans)	Homology modeling+ Molecular docking	Structure-guided mutagenesis screening to identify possible tethering sites (Cys mutants) & Rationalization of differential effect of trans and cis isomers	[9]
$GABA_AR$ (α1β2γ2L)	n.a.	Azogabazine	PTL	Antagonist (trans)	Homology modeling + Molecular docking	Rationalization of differential effect of trans and cis isomers & Design of validation mutagenesis	[10]
$GABA_AR$ (α1β3γ2)	β3(M283C) α1(V227C)	MAP20 (methanethio sulfonate+ azobenzene+ propofol)	PTL	Positive allosteric modulator (cis)	Homology modeling + Molecular docking	Identification of possible tethering sites (Cys mutants) & Rationalization of photomodulation differences depending on the Cys mutant	[11]

Table S3. (*cont.*) Computational studies of photoswitchable ligands targeting anionic pentameric ligand-gated ion channels.

Target protein	Tethering mutations	Photoswitchable ligand	Type	Activity	Computational methods	Aim	Reference
$GABA_AR$ (α1β2γ2) and $GABA_AR$-ρ (ρ1 and ρ2)	n.a.	Azo-NZ1	PCL	Pore blocker (trans)	Homology modeling + Molecular docking	Rationalization of the differential effect of trans and cis isomers, subtype selectivity and the effect of mutations	[12]
GlyR (α2 and α2β)	n.a.	azo-NZ1	PCL	Pore blocker (trans)	Homology modeling + Molecular docking	Rationalization of the differential effect of trans and cis isomers, subtype selectivity and the effect of mutations	[13]
GlyR (α2 and α2β)	n.a.	Glyght	PCL	Negative allosteric modulator (cis)	Homology modeling + Molecular docking	Rationalization of the differential effect of trans and cis isomers	[14]

Table S4. Computational studies of photoswitchable ligands targeting ionotropic glutamate receptors and ATP-dependent purinergic receptors.

**Target protein**	**Tethering mutations**	**Photoswitchable ligand**	**Type**	**Activity**	**Computational methods**	**Aim**	**Reference**
Kainate receptor (GluK2)	n.a.	gluazo (glutamate+ azobenzene)	PCL	Partial agonist (trans)	Experimental structure inspection + Manual docking	Rationalization of of the differential effect of trans and cis isomers	[15]
Kainate receptor (GluK2)	n.a.	gluazo (glutamate+ azobenzene)	PCL	Partial agonist (trans)	Molecular dynamics + Free energy calculations (US)	Rationalization of the differential effect of trans and cis isomers	[16]
Kainate receptor (GluR6)[i]	LiGluR6 (L439C) LiGluR6 (G486C)	MAG0 (maleimide+ azobenzene+ glutamate)	PTL[ii]	Agonist (cis or trans depending on the MAG compound)	Molecular dynamics + Free energy calculations (US)	Rationalization of the different isomer preference depending on the attachment Cys site	[17]
AMPA receptor (GluA2)	n.a.	ATA-3 compound (azobenzene+ tetrazolyl+ AMPA)	PCL	Agonist (trans)	Molecular docking + Molecular dynamics + Free energy calculations (US)	Rationalization of the differential effect of trans and cis isomers	[18]

[i] The glutamate ionotropic receptor kainate type subunit 2 is referred to as either GluR6 or GluK2; the latter is the recommended name in the UniProt database.

Table S4. (*cont.*) Computational studies of photoswitchable ligands targeting ionotropic glutamate receptors and ATP-dependent purinergic receptors.

**Target protein**	**Tethering mutations**	**Photoswitchable ligand**	**Type**	**Activity**	**Computational methods**	**Aim**	**Reference**
GluD receptor (GluD2)	LiGluD2 (I677C)	MAGu (maleimide+ azobenzene+ guanidinium)	PTL	Pore blocker	Homology modeling + Molecular covalent docking + Ion pore calculations	Structure-guided mutagenesis screening to identify possible tethering sites (Cys mutants) & Rationalization of the unusual pore blocking properties	[19]
P2X2 receptor	I336C N353C[ii]	MAM (maleimide+ azobenze+ maleimide)	PTL	Molecular tweezer (Cys-Cys crosslinker)	Molecular dynamics	Derivation of structural constraints to understand the (ATP-triggered) pore opening mechanism	[20]

[ii] Mutants considered in the MD simulations.

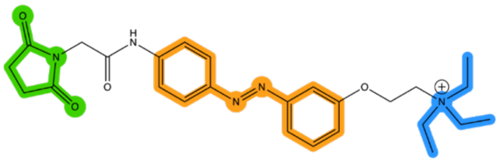

1: MAQ

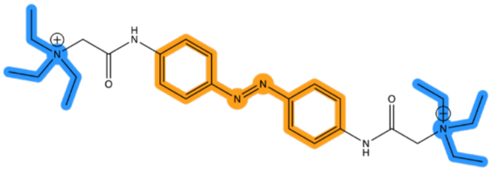

2: QAQ

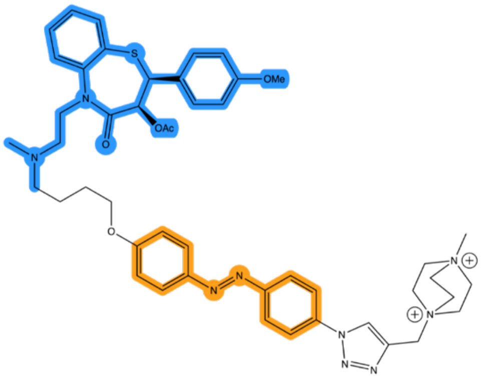

3: FHU-779

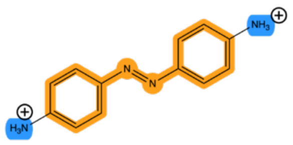

4: p-Diaminoazobenzene

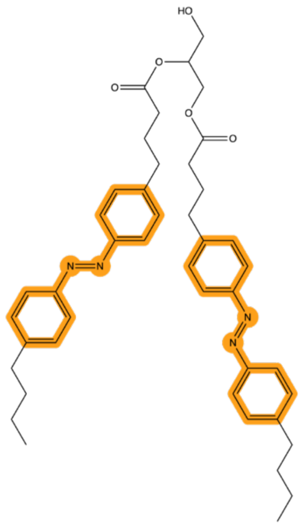

5: OptoDArG

Figure S1. Chemical structures of the PCLs and PTLs targeting voltage-gated ion channels mentioned in the text. The bioactive fragment is colored in blue, the photochromic group (azobenzene, shown in its *trans* form in the figure) in orange and the electrophilic group of PTLs in green.

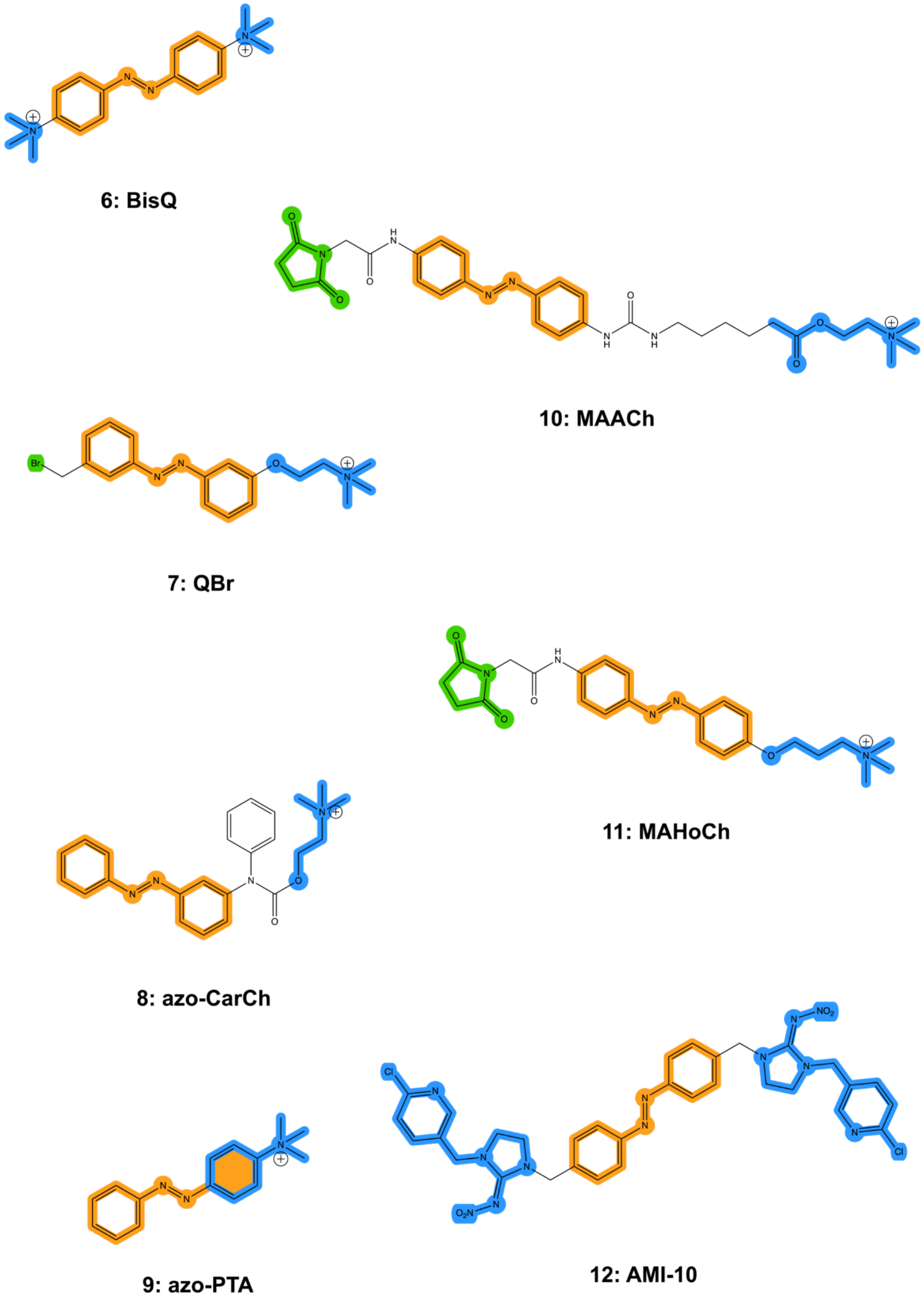

Figure S2. Chemical structures of the PCLs and PTLs targeting cationic pentameric ligand-gated ion channels. The same coloring code as in Figure S1 is used. Note that the design of azo-PTA (**9**) capitalizes on the phenyl group already present in the bioactive group (phenyltrimetylammonium) to integrate one of the phenyl rings of the azobenzene photochromic group.

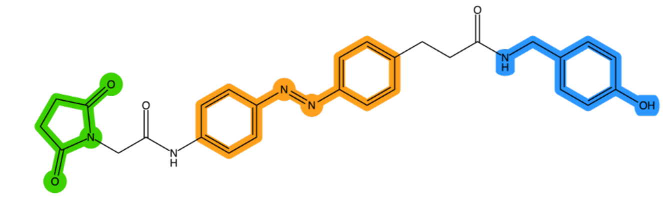

13: MAB-0

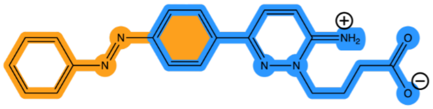

14: Azogabazine

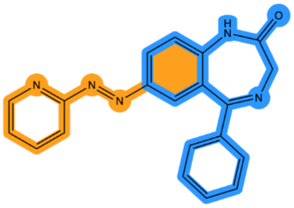

17: Glyght

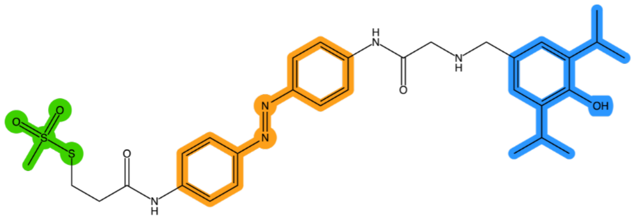

15: MAP20

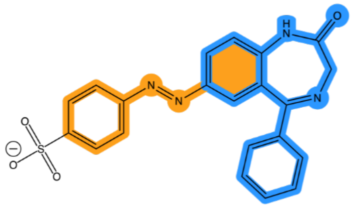

16: Azo-N1

Figure S3. Chemical structures of the PCLs and PTLs targeting anionic pentameric ligand-gated ion channels. The same coloring code as in Figure S1 is used. Note that the design of azogabazine (**14**), azo-NZ1 (**16**) and Glyght (**17**) capitalize on the phenyl group already present in the bioactive group (gabazine for **14** and nitrazepam for **16** and **17**) to integrate one of the phenyl rings of the azobenzene photochromic group.

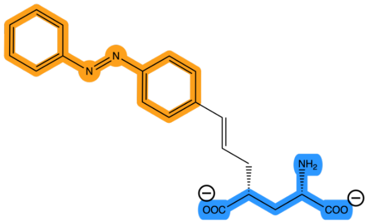

18: 4-GluAzo

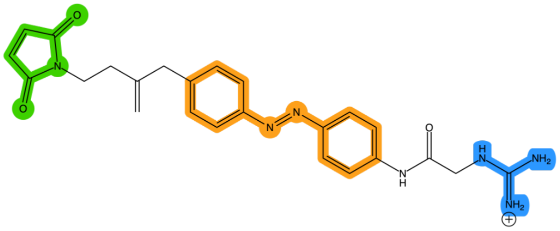

21: MAGu

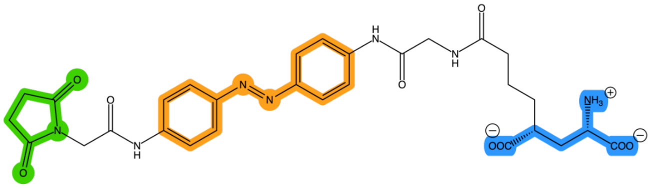

19: MAG0

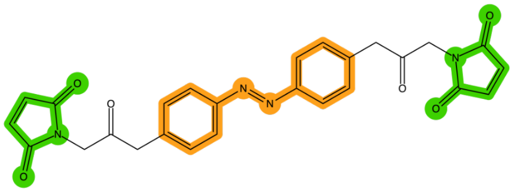

22: MAM

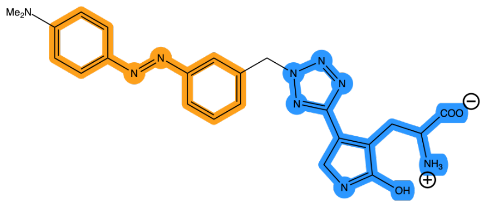

20: ATA-3

Figure S4. Chemical structures of the PCLs and PTLs targeting ionotropic glutamate receptors and ATP-dependent purinergic receptors. The same coloring code as in Figure S1 is used.

References

1. Mourot, A.; Herold, C.; Kienzler, M. A.; Kramer, R. H. Understanding and improving photocontrol of ion channels in nociceptors with azobenzene photo- switches. *Brit. J. Pharmacol.* **2018**, *175*(12), 2296-2311.

2. Fehrentz, T.; Huber, F. M.; Hartrampf, N.; Bruegmann, T.; Frank, J. A.; Fine, N. H.;Malan, D.; Danzl, J.G.; Tikhonov, D. B.; Sumser, M.; Sasse, P.; Hodson, D.J.; Zhorov, B.S., Klöcker, N.; Trauner, D. Optical control of L-type Ca^{2+} channels using a diltiazem photoswitch. Nature Chem. Biol. **2018**, 14(8), 764-767.

3. Palmisano, V.F.; Gómez-Rodellar, C.; Pollak, H.; Cárdenas, G.; Corry, B.; Faraji, S.; Nogueira, J.J. Binding of azobenzene and p-diaminoazobenzene to the human voltage-gated sodium channel Nav1.4. *Phys. Chem. Chem. Phys.* **2021**, *23*(5), 3552-3564.

4. Lichtenegger, M.; Tiapko, O.; Svobodova, B.; Stockner, T.; Glasnov, T.N.; Schreibmayer, W.; Platzer, D.; de la Cruz, G.G.; Krenn, S.; Schober, R.; Shrestha, N. An optically controlled probe identifies lipid-gating fenestrations within the TRPC3 channel. *Nature Chem. Biol.* **2018**, *14*(4), 396-404.

5. Tochitsky, I.; Banghart, M.R.; Mourot, A.; Yao, J.Z.; Gaub, B.; Kramer, R.H.; Trauner, D. Optochemical control of genetically engineered neuronal nicotinic acetylcholine receptors. *Nature Chem.* **2012**, *4*(2), 105-111.

6. Xu, Z.; Shi, L.; Jiang, D.; Cheng, J.; Shao, X.; Li, Z. Azobenzene modified imidacloprid derivatives as photoswitchable insecticides: Steering molecular activity in a controllable manner. *Sci. Rep.* **2015**, *5*(1), 1-8.

7. Melis, C.; Bussi, G.; Lummis, S.C.; Molteni, C. Trans−cis switching mechanisms in proline analogues and their relevance for the gating of the 5-HT3 receptor. *J. Phys. Chem. B* **2009**, *113*(35), 12148-12153.

8. Crnjar, A.; Comitani, F.; Hester, W.; Molteni, C. Trans–cis proline switches in a pentameric ligand-gated ion channel: how they are affected by and how they affect the biomolecular environment. *J. Phys. Chem. Lett.* **2019**, *10*(3), 694-700.

9. Lin, W.C.; Davenport, C.M.; Mourot, A.; Vytla, D.; Smith, C.M.; Medeiros, K.A.; Chambers, J.J.; Kramer, R.H. Engineering a light-regulated $GABA_A$ receptor for optical control of neural inhibition. *ACS Chem. Biol.* **2014**, *9*(7), 1414-1419.

10. Mortensen, M.; Huckvale, R.; Pandurangan, A.P.; Baker, J.R.; Smart, T.G. Optopharmacology reveals a differential contribution of native $GABA_A$ receptors to dendritic and somatic inhibition using azogabazine. *Neuropharmacology* **2020**, *176*, 108135.

11. Borghese, C.M.; Wang, H.Y.L.; McHardy, S.F.; Messing, R.O.; Trudell, J.R.; Harris, R.A.; Bertaccini, E.J. Modulation of α1β3γ2 $GABA_A$ receptors expressed in *X. laevis* oocytes using a propofol photoswitch tethered to the transmembrane helix. *Proc. Natl. Acad. Sci. USA* **2021**, *118*(8), e2008178118.

12. Maleeva, G.; Wutz, D.; Rustler, K.; Nin- Hill, A.; Rovira, C.; Petukhova, E.; Bautista-Barrufet, A.; Gomila- Juaneda, A.; Scholze, P.; Peiretti, F.; Alfonso- Prieto, M.; König, B.; Gorostiza, P.; Bregestovski, B. A photoswitchable GABA receptor channel blocker. *Brit. J. Pharmacol.* **2019**, *176*(15), 2661-2677.

13. Maleeva, G.; Nin-Hill, A.; Rustler, K.; Petukhova, E.; Ponomareva, D.; Mukhametova, E.;

Gomila, A.M.; Wutz, D., Alfonso-Prieto, M.; König, B.; Gorostiza, P. Subunit-specific photocontrol of glycine receptors by azobenzene-nitrazepam photoswitcher. *eNeuro* **2021**, *8*(1), ENEURO.0294-20.2020.

14. Gomila, A.M.; Rustler, K.; Maleeva, G.; Nin-Hill, A.; Wutz, D.; Bautista-Barrufet, A.; Rovira, X.; Bosch, M.; Mukhametova, E.; Petukhova, E.; Ponomareva, D. Photocontrol of endogenous glycine receptors in vivo. *Cell Chem. Biol.* **2020** *27*(11), 1425-1433.
15. Reiter, A.; Skerra, A.; Trauner, D.; Schiefner, A.. A photoswitchable neurotransmitter analogue bound to its receptor. *Biochemistry* ,**2013** *52*(50), 8972-8974.
16. Guo, Y.; Wolter, T.; Kubař, T.; Sumser, M.; Trauner, D.; Elstner, M. Molecular dynamics investigation of *gluazo*, a photo-switchable ligand for the glutamate receptor GluK2. *PLOS One* **2015**, *10*(8), e0135399.
17. Numano, R.; Szobota, S.; Lau, A.Y.; Gorostiza, P.; Volgraf, M.; Roux, B.; Trauner, D.; Isacoff, E.Y. Nanosculpting reversed wavelength sensitivity into a photoswitchable iGluR. *Proc. Natl. Acad. Sci. USA* **2009**, *106*(16), 6814-6819.
18. Wolter, T.; Steinbrecher, T.; Trauner, D.; Elstner, M. Ligand photo-isomerization triggers conformational changes in iGluR2 ligand binding domain. *PLOS One* **2014**, *9*(4), e92716.
19. Lemoine, D.; Mondoloni, S.; Tange, J.; Lambolez, B.; Faure, P.; Taly, A.; Tricoire, L.; Mourot, A. Probing the ionotropic activity of glutamate GluD2 receptor in HEK cells with genetically-engineered photopharmacology. *eLife* **2020**, *9*, p.e59026.
20. Habermacher, C.; Martz, A.; Calimet, N.; Lemoine, D.; Peverini, L.; Specht, A.; Cecchini, M.; Grutter, T. Photo-switchable tweezers illuminate pore-opening motions of an ATP-gated P2X ion channel. *eLife* **2016**, *5*, e11050.
